# A Novel Laboratorial Approach to Evaluate Bacterial Microleakage of Endodontic Sealers

**DOI:** 10.1002/cpz1.70407

**Published:** 2026-07-01

**Authors:** Emannuel Fonseca Thomé da Silva Monteiro, Alice Abib Ramos Fabri, Marcelo Barbosa Garcia, Sabrina Silva Azevedo, Higor Machado Gonçalves, Ana Clara Miranda Porto Figueiredo, Fabrícia Nogueira Klein, Gabriela Ceccon Chianca, Roberta Barcelos, Helvécio Cardoso Corrêa Póvoa, Leonardo Santos Antunes, Natalia Lopes Pontes Póvoa Iorio

**Affiliations:** ^1^ Experimental and Applied Microbiology Laboratory Nova Friburgo Institute of Health, Universidade Federal Fluminense (UFF) Nova Friburgo Rio de Janeiro Brazil; ^2^ Graduate Program in Dentistry Nova Friburgo Institute of Health, UFF Nova Friburgo Rio de Janeiro Brazil; ^3^ Graduate Program in Pathology School of Medicine, UFF Niterói Rio de Janeiro Brazil; ^4^ Department of Specific Formation Nova Friburgo Institute of Health, UFF Nova Friburgo Rio de Janeiro Brazil; ^5^ Graduate Program in Applied Microbiology and Parasitology Biomedical Institute, UFF Niterói Rio de Janeiro Brazil; ^6^ Graduate Program in Dentistry School of Dentistry, UFF Niterói Rio de Janeiro Brazil

**Keywords:** bacteria, dental leakage, endodontics, endodontic sealer, microbiology

## Abstract

To avoid failure of endodontic treatment, it is important to properly seal the root canals after chemomechanical preparation in order to avoid microleakage. Typically, sealing involves the use of gutta‐percha and sealing cement, the latter of which adheres to the dentin and fills the spaces between the gutta‐percha and the dentin walls. Laboratory studies have been performed to detect microleakage of endodontic sealers based on various criteria, including microbiological ones; however, none guaranteed that the leakage occurred through the root canal space and not through other pathways. This article proposes a new laboratory assay to evaluate bacterial microleakage in endodontic sealers, ensuring visualization of the correct inoculum infiltration pathway. Furthermore, we present the main critical parameters and provide suggestions on how to manage them. These protocols allow the detection of bacterial microleakage in bovine teeth filled with endodontic sealer and inoculated with *Enterococcus faecalis*. © 2026 The Author(s). *Current Protocols* published by Wiley Periodicals LLC.

**Basic Protocol 1**: Bovine teeth selection and cleaning

**Basic Protocol 2**: Bovine teeth inclusion

**Basic Protocol 3**: Tooth standardization

**Basic Protocol 4**: Set preparation

**Basic Protocol 5**: Inoculum preparation

**Basic Protocol 6**: Canal inoculation

**Basic Protocol 7**: Set incubation

## INTRODUCTION

The tooth has two main parts, the crown and root, externally covered by enamel and cement, respectively. Internally, it is composed of dentin and dental pulp (Farci & Soni, 2023). Endodontic infection occurs mainly due to exposure of the dental pulp to the oral cavity (Nair, 2004), and the presence of microorganisms in the root canal system triggers an inflammatory response resulting in apical periodontitis (Aw, 2016; Kakerashi et al., 1965; Nair, 2004).

The success of endodontic treatment in teeth with apical periodontitis is associated with reducing the bacterial load to levels compatible with healing (Siqueria & Roças, 2008). This treatment relies on a chemomechanical approach, which combines instrumentation and irrigation procedures for achieving effective cleansing of the root canal system (Byström et al., 1987; Garcez et al., 2007). To avoid endodontic treatment failure, it is also important to properly seal the canals after chemomechanical preparation in order to prevent microleakage (Deshpande & Naik, 2015; Jafari & Jafari, 2017, Komabayashi et al., 2020; Muliyar et al., 2014).

Typically, sealing involves the use of gutta‐percha, a semi‐solid material, and sealing cement. Gutta‐percha acts as a filling material, whereas endodontic cement adheres to the dentin and fills spaces between the gutta‐percha and the dentinal walls (Muliyar et al., 2014).

Endodontic sealers are classified by composition as zinc oxide–eugenol, salicylate, zinc oxide–fatty acid, glass ionomer, silicone, epoxy resin, tricalcium silicate (MTA, or mineral trioxide aggregate/bioceramic), and methacrylate resin (Komabayashi et al., 2020). To date, different laboratory studies have been performed to detect microleakage of endodontic sealers, based on dye penetration, dye extraction, fluid filtration, glucose penetration, bacterial leakage, endotoxin infiltration, caffeine penetration, caffeine microleakage, protein penetration, copper ion diffusion, radioisotope penetration, and electrochemical leakage tests (Jafari & Jafari, 2017; Komabayashi et al., 2020). Review studies have described limitations and disadvantages associated with these methodologies (Jafari & Jafari 2017; Rechenberg et al., 2011; Verissimo & Do Vale, 2006), especially regarding microbial leakage in two‐chamber models. The main limitation they report is that these models do not guarantee that the leakage actually occurred through the root canal space pathways and not through other pathways (Rechenberg et al., 2011).

**Figure 1:**
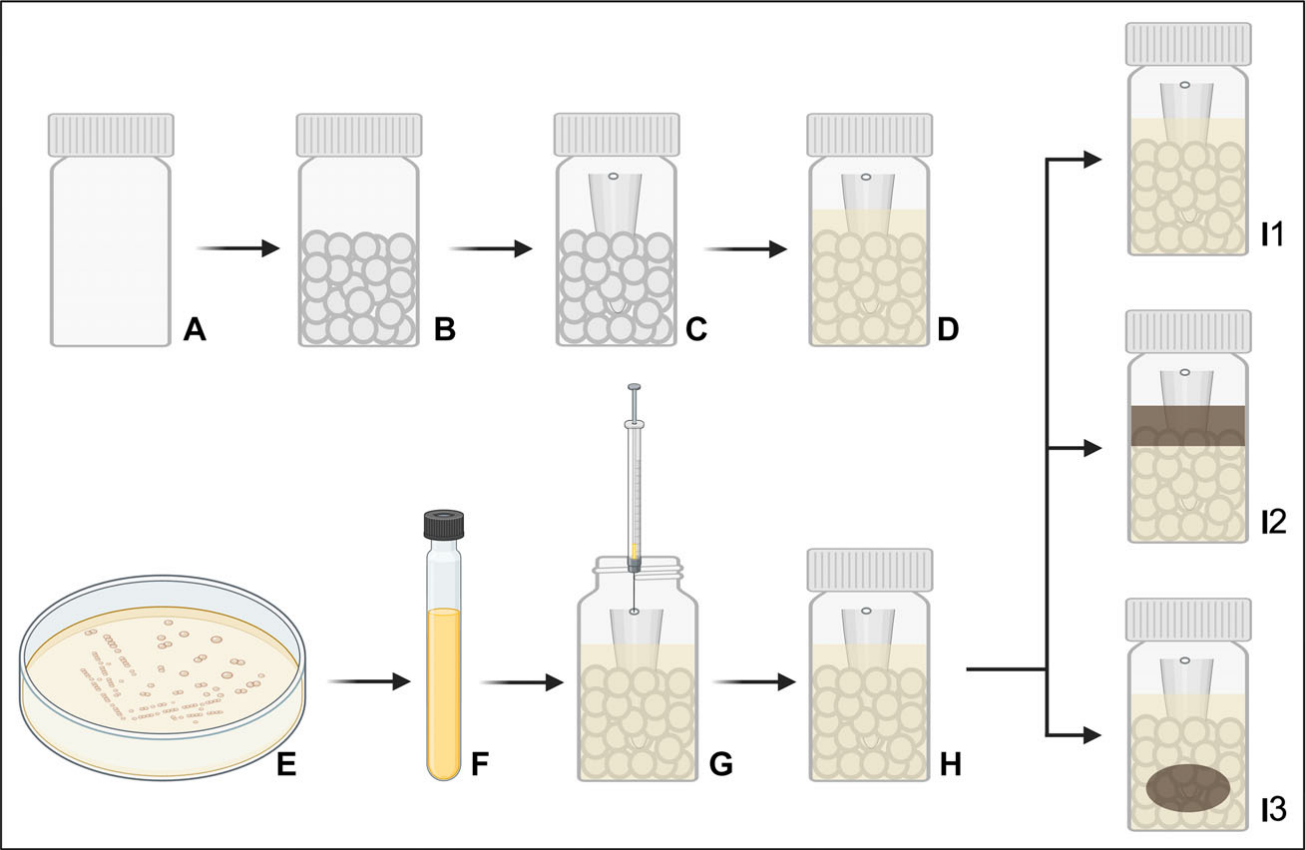
Novel laboratory approach to evaluate the bacterial microleakage of endodontic sealers. (**A**) Glass tube with a lid. (**B**) Glass pearls inserted inside the tube. (C) Obturated bovine root within the glass pearls. (**D**) Addition of bile aesculin agar to the tube. (**E**) *E. faecalis* ATCC 29212 added to agar culture medium. (**F**) *E. faecalis* in broth culture medium. (**G**) Root inoculation. (**H**) Inoculated root. (**I**) Main possible results after incubation (I1, no bacterial microleakage; I2, visualization of contamination by the external root pathway; I3, visualization of bacterial microleakage by the obturation pathway). Created via BioRender.com/4bx5yxs.

Our article describes a novel laboratory assay to evaluate the bacterial microleakage of endodontic sealers (Fig. 1), ensuring visualization of the correct inoculum leakage pathway. This allows identification of whether the leakage is occurring through the root canal space. We describe the selection and cleaning of bovine teeth in Basic Protocol 1. Basic Protocol 2 presents the inclusion criteria and storage of the teeth. Once teeth are included, the decoronation process and standardization of shape and size of the root canals are described in Basic Protocol 3, and Basic Protocol 4 details the preparation of a set containing the tooth to be inoculated. The inoculum preparation and the set inoculation with *Enterococcus faecalis* are described in Basic Protocols 5 and 6, respectively. Basic Protocol 7 demonstrates how to incubate and maintain the sets throughout all 6 weeks of the experiment.

## STRATEGIC PLANNING

Obtaining the samples of freshly extracted bovine teeth (Basic Protocol 1) must be carefully planned prior to the experiment. This will depend on previous contact with a slaughterhouse so that they can plan and reserve the number of teeth requested.

Due to the limited availability of human teeth (Tanaka et al., 2008) and the greater accessibility of bovine teeth (Schmalz et al., 2001), bovine ones are used in these protocols. The use of bovine teeth in endodontic research has increased considerably and has demonstrated satisfactory applicability (Azevedo et al., 2025; Brackett et al., 1998; de Carvalho et al., 2018; Schmalz et al., 2001; Silva et al., 2019; Yassen et al., 2011). Although bovine dentin presents greater permeability and higher density, the number and diameter of dentinal tubules are similar to those of human dentin (Schilke et al., 2000), making bovine teeth a viable alternative for permeability and bond strength tests involving gutta‐percha and sealer obturation (de Carvalho et al., 2018; Silva et al., 2019).

Obtaining the reference strain, *E. faecalis* American Type Culture Collection (ATCC) 29212 (Basic Protocol 5), must be planned prior to the experiment.

*E. faecalis* was selected due to its frequent association with persistent endodontic infections, its ability to form biofilms, its resistance to antimicrobial agents and disinfection procedures, its survival under adverse environmental conditions, and its tolerance to high pH levels (Nakajo et al., 2006; Prada et al., 2019; Stuart et al., 2006). Additionally, it is important to highlight that this species, particularly the reference strain ATCC 29212, is the most commonly used microorganism in experimental endodontic laboratory studies (Oishi et al., 2026; Swimberghe et al., 2019).

*CAUTION: E. faecalis* is a Biosafety Level 2 (BSL‐2) pathogen. Follow all appropriate guidelines and regulations for the use and handling of pathogenic microorganisms. The use of personal protective equipment (PPE), like a lab coat, gloves, and a face shield and mask, during all protocols is essential to avoid contamination of the operator and the sample. Microbiological steps should be performed in an aseptic environment such as a biosafety cabinet (according to the manufacturer's instructions) or working area of the Bunsen burner to avoid contamination.

*NOTE*: Dental procedures should be performed by a specialist in the field. It is important to highlight that procedures involving root canals must be performed by a specialist in endodontics. Microbiological procedures must be performed by a microbiology specialist.

## Basic Protocol 1 BOVINE TEETH SELECTION AND CLEANING

Basic Protocol 1 describes a method to select bovine teeth by visual inspection and then clean them using a periodontal curette. This protocol allows the selection of clean bovine teeth with completely formed roots and a root size of ≥22 mm.

### Materials

Fresh bovine teeth
0.9% (w/v) saline, sterile (see recipe)

Face shield
Periodontal Gracey curette (Golgran‐Millennium)
Plastic flask with lid

*NOTE*: Obtain fresh bovine teeth from a slaughterhouse after the animal has been slaughtered (a routine activity in the commercialization of meat for nutritional purposes). The Brazilian Council for the Control of Animal Experimentation, as well as the Institutional Animal Care and Use Committee (IACUC), only evaluates the use of live animals in research or animals that have been euthanized for research purposes, which is not the case in this protocol, so ethical approval is not required here. Wear gloves to avoid contamination when handling these teeth, as fresh bovine teeth routinely come with biological materials.

#### Visual evaluation

**Figure 2:**
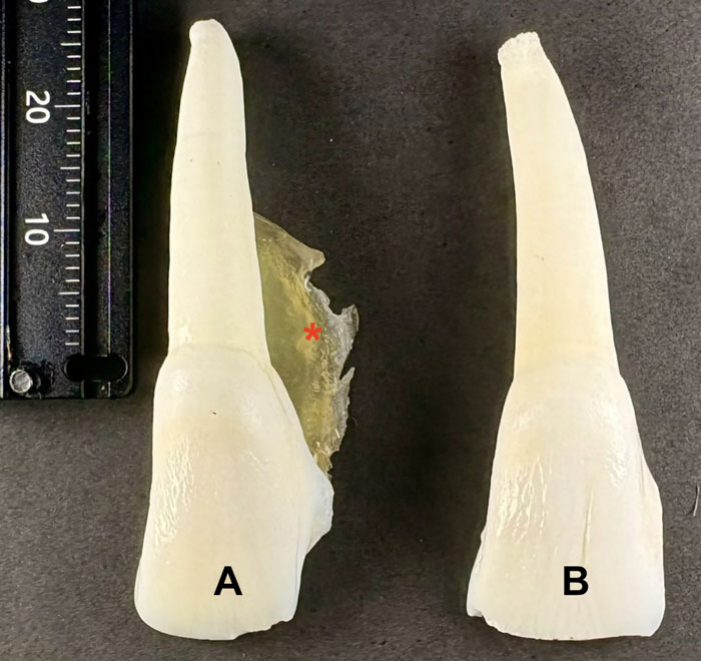
Selection of bovine teeth with fully formed roots and a minimum length of 22 mm. (**A**) Bovine tooth showing external structures, including an amorphous yellow structure (indicated by a red asterisk). This external structure must be removed prior to use in the study. (**B**) Clean bovine tooth with no external structures.

1 Perform a visual inspection of the fresh bovine teeth. Select bovine teeth with completely formed roots, without cracks, and a root size of ≥22 mm (Fig. 2).
  It is necessary to be in a well‐lit environment to ensure visual acuity.

#### Cleaning

2 Wear a face shield to protect the face.
  A face shield is necessary to protect against bone fragments and calculus from the bovine teeth that might detach during the cleaning process.
3 Remove any external teeth structures, such as bone and calculus (Fig. 2A), using a periodontal Gracey curette.
4 Store clean teeth (Fig. 2B) in sterile 0.9% saline in a plastic flask with a lid in a refrigerator at 4°C.
  Replace the sterile saline solution once a week.

## Basic Protocol 2 BOVINE TEETH INCLUSION

Basic Protocol 2 describes a method to determine which teeth to include and how to store them. Radiographic evaluation with DIOX® intraoral portable X‐ray (Micro Imagem Industry Commerce Import and Export) and exposure using a digital phosphor plate system with VistaScan Nano View (Durr Dental) allow the inclusion of bovine teeth with a single root canal from among those initially selected in Basic Protocol 1.

### Materials

Cleaned bovine teeth (see Basic Protocol 1)
0.9% (w/v) saline, sterile (see recipe)

X‐ray protective clothing
PPE
Utility wax (Lysanda Dental Products)
#15 blade
Phosphor plate digital system (Durr Dental)
DIOX® intraoral portable X‐ray (Micro Imagem Industry Commerce Import and Export)
VistaScan Nano Easy digital system connected to notebook
Vista Soft 3.0 software (Durr Dental)
Plastic flask with lid

#### Radiographic evaluation

**Figure 3:**
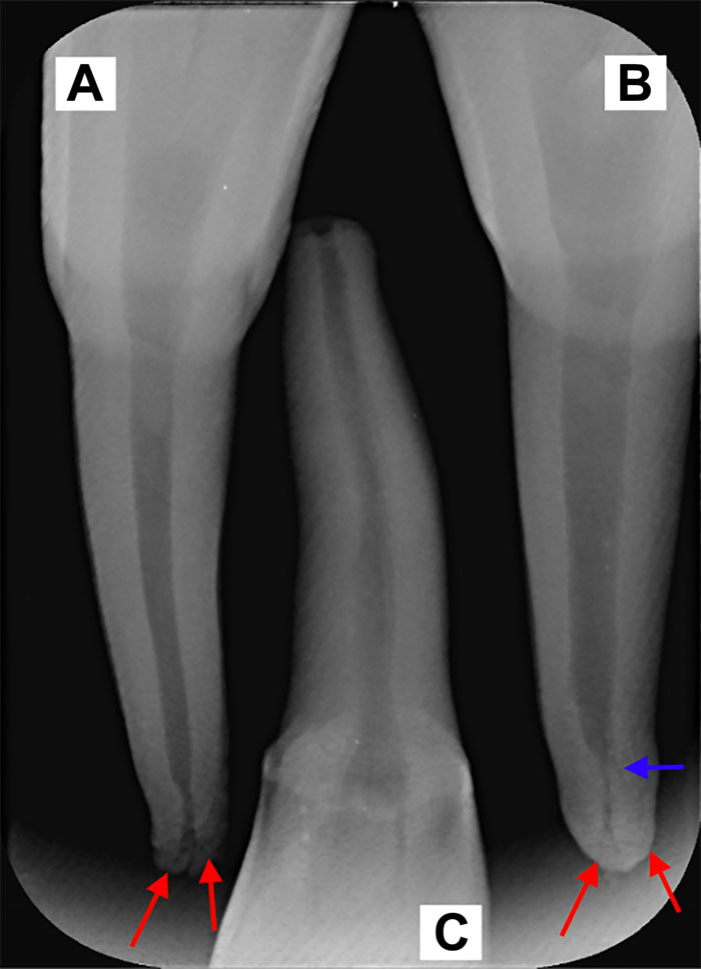
Digital image of bovine teeth. (**A**) Excluded tooth due to apical delta. (**B**) Excluded tooth due to lateral canals and apical delta. (**C**) Included tooth presenting a single root canal. Red arrows indicate apical delta, and the blue arrow indicates lateral canals.

1 Wear X‐ray protective clothing and PPE.
  The use of radiographic protective clothing is necessary to avoid exposing the operator to secondary radiation from the X‐ray machine.
2 Cut 4 × 1–cm piece of utility wax using a #15 blade.
  It is not necessary to use a handle for the scalpel blade because the wax is soft.
3 Position the utility wax on the long axis of the phosphor plate digital system on a flat horizontal surface.
4 Place a cleaned bovine tooth on the utility wax so that it is on its side on top of the phosphor plate.
5 Sensitize the phosphor plate by positioning the DIOX® intraoral portable X‐ray at a distance of 10 cm from the phosphor plate, with 35‐s exposure.
6 Insert the phosphor plate into the VistaScan Nano Easy digital system connected to a notebook to produce a digital image in Vista Soft 3.0 software.
7 Exclude teeth with multiple root canals, lateral canals, or apical deltas (Fig. 3A and 3B).

8 Include only bovine teeth with a single root canal (Fig. 3C).

#### Bovine teeth storage

9 Fill a plastic flask with sterile 0.9% saline.
10 Put the included teeth in the flask and close the lid.
11 Store the flask containing the included teeth in a refrigerator at 4°C.
  Replace the sterile saline solution once a week.

## Basic Protocol 3 TOOTH STANDARDIZATION

To obtain a standardized and clean sample of bovine roots, Basic Protocol 3 describes the decoronation process, standardization regarding shape and size, and isolation and sterilization of the root canals.

### Materials

Included bovine teeth (see Basic Protocol 2)
2.5% (w/v) sodium hypochlorite (NaOCl)
17% (w/v) ethylenediaminetetraacetic acid (EDTA; Lysanda Dental Products)

PPE
Double‐sided diamond cutting disc (Microdont)
Mandrel clamping device (Microdont)
Dental laboratory electric motor, with suspension engine (Metalurgica FAVA Industria e Comercio)
Endodontic ruler
Permanent marker
Bench vise
10‐ml syringes with needles
#10 and #15 files (K‐file #10 and K‐file #15, Dentsply Sirona)
#3, #4, and #5 Largo drills (Dentsply Sirona)
VDW Silver Reciproc® electric motor (Dentsply Sirona), with contra‐angle
#20.07, #25.07, #35.06, and #45.05 mechanized reciprocating files (W File #20.07, TDK, Shenzhen Superline Technology)
Utility wax (Lysanda Dental Products)
Easy Clean instrument (Bassi Equipamentos)
White nail polish
Autoclave
Surgical‐grade pack

#### Bovine tooth decoronation

1 Wear PPE to avoid accidents and contamination.
*CAUTION*: It is important to use PPE at this stage due to the formation of dentin dust during decoronation, which can lead to eye irritation and airway dryness.
2 Connect the double‐sided diamond cutting disc on the mandrel clamping device and attach them to the straight handpiece of the dental laboratory electric motor.
3 Cut the crown of each included bovine tooth 2 to 3 mm below the cemento‐enamel junction.

#### Standardization of the bovine root's cervical third

4 Standardize all roots by using an endodontic ruler and a permanent marker to trace a 22‐mm distance from the apical region to the cervical region of each root and cut at this mark.
5 Insert the bovine tooth in the bench vise and fill it with 10 ml of 2.5% NaOCl using a 10‐ml syringe with needle.
6 Introduce a #10 file into the root canal with oscillatory and passive cyclic motions (clockwise to fit the canal wall and counterclockwise to cut with controlled pressure). During this process, irrigate the canal with 2.5% NaOCl using the 10‐ml syringe with the needle attached, before each cycle and between cycles, to remove debris until #10 file reaches the apex (working length).
7 Repeat exactly the same kinematics presented in step 6 with a #15 file.
8 Connect a #3 Largo drill on the oscillating contra‐angle of the VDW Silver Reciproc® electric motor.
9 Enlarge the cervical preflaring of the root with the #3 Largo drill in continuous rotation at 1000 rpm.
  Insert only the active part of the Largo drill in the canal.
10 Repeat steps 8 and 9 with a #4 Largo drill.
11 Repeat steps 8 and 9 with a #5 Largo drill.

#### Standardization of the bovine root's third medium apex

12 Connect the #20.07 mechanized reciprocating file on the oscillating contra‐angle of the VDW Silver Reciproc® electric motor.
13 Gently introduce the file into the root canal, advancing apically with a pecking (descending and ascending) motion in sets of three pecks. Repeat this step until the apex is reached. During this process, irrigate the channel with 2.5% NaOCl, before each set of three pecks and between sets, to remove debris. After each set of three pecks, remove the instrument from the root canal and clean the file.
14 Repeat steps 12 and 13 with a #25.07 mechanized reciprocating file.
15 Repeat steps 12 and 13 with a #35.06 mechanized reciprocating file.
16 Repeat steps 12 and 13 with a #45.05 mechanized reciprocating file.

#### Cleansing of the bovine root canal

17 Close the apex of the root temporarily using the smallest possible amount of utility wax.
  This step allows the irrigating substances to remain in the canal during the cleansing process.
18 Fill a 10‐ml syringe with 17% EDTA and another 10‐ml syringe with 2.5% NaOCl.
19 Gently irrigate the bovine root canal with 2 ml of 17% EDTA.
20 Use the Easy Clean instrument attached to the contra‐angle of the VDW Silver Reciproc® electric motor, in continuous rotation at 1000 rpm for 20 s, to activate the EDTA.
21 Gently irrigate the bovine root canal with 2 ml of 2.5% NaOCl.
22 Use the Easy Clean instrument attached to the contra‐angle of the VDW Silver Reciproc® electric motor, in continuous rotation at 1000 rpm for 20 s, to activate the NaOCl.
23 Repeat steps 19 through 22 two more times.
  Steps 5 to 23 comprise the endodontic chemomechanical preparation.

#### Sealing and sterilizing the bovine teeth

24 After cleaning the bovine root canals, leave them at room temperature for 24 hr.
  It is important to wait 24 hr so that the bovine rennet can dry completely.
25 Apply two coats of white nail polish to all root surfaces except the apex and on the coronal plane. Wait 24 hr between each application of nail polish.
  This step allows us to isolate possible cracks and side channels on the external root that were not previously observed.
26 Twenty‐four hours after the second layer of enamel has dried, sterilize the bovine roots by autoclaving using a surgical‐grade pack for each root.

## Basic Protocol 4 SET PREPARATION

Basic Protocol 4 describes the method for obtaining sterile sets composed of glass pearls, a bovine root filled with the test endodontic sealer, and solid culture medium in a glass tube with a lid. Although this protocol does not describe the process of obturation with endodontic sealers, we strongly recommend that this process be performed strictly following the manufacturer's instructions.

### Materials

Glass pearls (3 mm)
Bile aesculin agar medium (see recipe)
Standardized, cleaned bovine teeth (see Basic Protocol 3)
Endodontic sealer being tested
17% (w/v) EDTA (Lysanda Dental Products)

Analytical balance
5‐ml borosilicate glass tubes (16 × 41–mm) with lids
Autoclave
Surgical‐grade pack
100‐ml borosilicate glass flask with lid
60°C water bath
#4 Largo drill (Dentsply Sirona)
Easy Clean instrument (Bassi Equipamentos)
VDW Silver Reciproc® electric motor (Dentsply Sirona), with contra‐angle
Tweezers, sterilized
Micropipet with sterile micropipet tips

**Figure 4:**
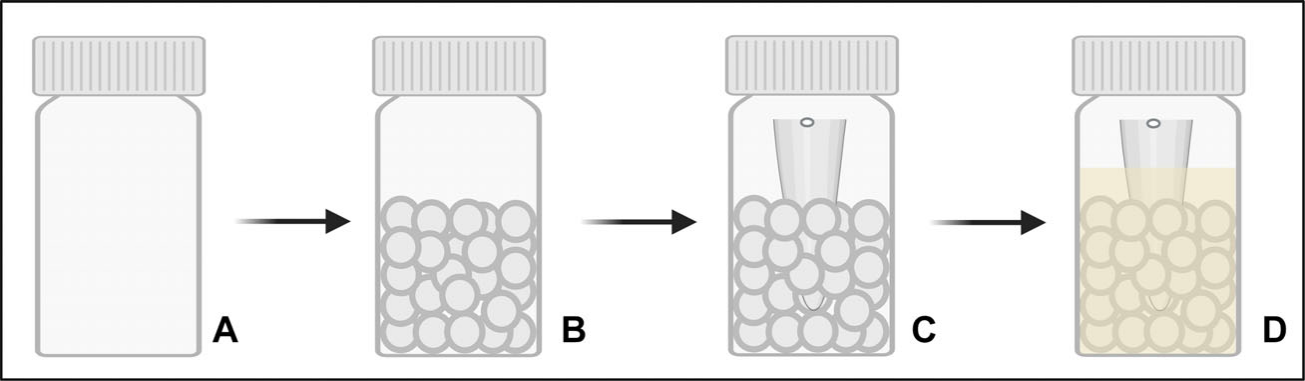
Set preparation. (**A**) Empty 5‐ml borosilicate glass tube (16 × 41 mm) with a lid. (**B**) A total of 3.5 g glass pearls inside the tube. (**C**) Bovine root immediately after obturation with test sealer inserted within the glass pearls 5 mm short of the bottom of the tube. (**D**) Full set, composed of the root, glass pearls, and 1.6 ml bile aesculin agar medium inside the glass tube with a lid. Created via BioRender.com/1j7mipz.

1 Weigh 3.5 g glass pearls.
2 Place the pearls (Fig. 4B) in a 5‐ml borosilicate glass tube with a lid (Fig. 4A).

3 Autoclave (121°C/20 min) the tube of glass pearls in a surgical‐grade pack.
4 Place the bile aesculin agar medium, in a 100‐ml borosilicate glass flask with a lid, in a 60°C water bath.
  The water bath is important to keep the bile aesculin agar medium between 55° and 60°C, allowing the medium to remain in the liquid state after the autoclaving process.
5 Perform the root obturation on the standardized, cleaned bovine teeth with the endodontic sealer being tested, strictly following the manufacturer's instructions. After obturation, remove the excess endodontic sealer using a #4 Largo drill, keeping 3 mm of obturation. Remove the smear layer with 17% EDTA under agitation using the Easy Clean instrument attached to the contra‐angle of the VDW Silver Reciproc® electric motor, in continuous rotation at 1000 rpm for 20 s.
*IMPORTANT NOTE*: Perform step 5 using aseptic techniques in an aseptic environment.
6 Using sterilized tweezers, insert each root (immediately after root canal obturation with the test sealer) aseptically within the autoclaved glass pearls (see step 3) 5 mm short of the bottom of the 5‐ml glass tube (Fig. 4C).
  Make sure the tubes with glass pearls are completely dry before performing step 6.
*IMPORTANT NOTE*: To avoid contamination, steps 6 and 7 need to be performed using aseptic techniques [wear PPE and disinfect gloves and surfaces with 70% (v/v) ethanol] in an aseptic environment such as a biosafety cabinet, laminar flow hood, or working area of a Bunsen burner.
7 Using a sterile micropipet tip, aseptically and gently insert 1.6 ml (2 × 800 µl) of the bile aesculin agar medium (at 55°C to 60°C; see step 4) into the 5‐ml glass tube (Fig. 4D).
  The sterile tip (previously attached to the micropipet) should be leaned against the internal wall of the 5‐ml tube to avoid touching the root. Do not move the tube until the medium solidifies.
8 Set the bacteriological incubator to 36°C and incubate the sets (tubes containing root, glass pearls, and medium) for 24 hr before inoculating them (see Basic Protocol 5).
  This 24‐hr period is important to observe any contamination that might have occurred during set preparation as well as to allow hardening of the endodontic sealer.

## Basic Protocol 5 INOCULUM PREPARATION

This protocol describes how to obtain an inoculum with 10^9^ colony‐forming units (CFU)/ml of *E. faecalis* ATCC 29212, from a frozen glycerol stock, in brain‐heart infusion (BHI) broth.

### Materials

*E. faecalis* ATCC 29212 frozen stock, in BHI broth with 20% (v/v) glycerol
BHI agar plate, sterile (see recipe)
BHI broth medium, sterile (see recipe)

Inoculation loops, sterile
36°C bacteriological incubator
13 × 100–mm glass tubes
Spectrophotometer (Libra S2 Colorimeter, Biochrom)

**Figure 5:**
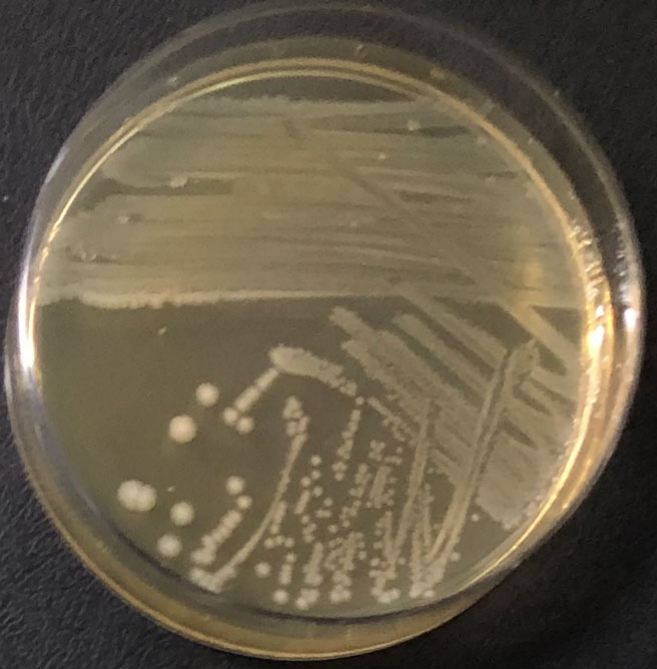
*E. faecalis* ATCC 29212 on BHI agar after incubation at 36°C for 24 hr.

**Figure 6:**
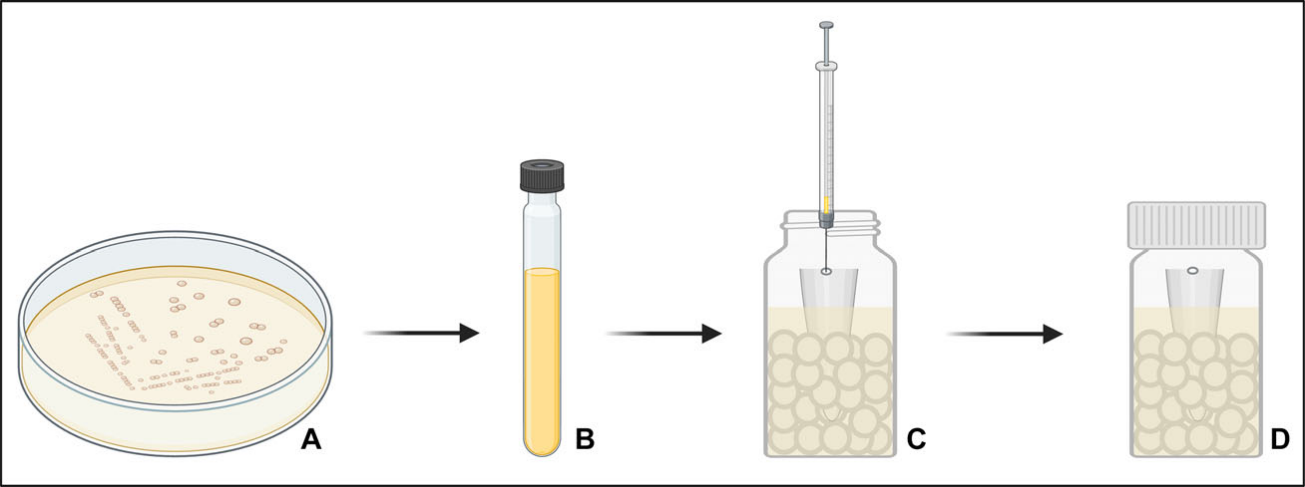
Root canal inoculation. (**A**) *E. faecalis* ATCC 29212 grown on BHI agar after incubation at 36°C for 24 hr. (**B**) Inoculum of *E. faecalis* in BHI broth. (**C**) Inoculation process using a sterile syringe with a needle. (**D**) Set containing the inoculated root. Created via BioRender.com/wr26vyl.

1 Using aseptic technique, remove a small chunk of *E. faecalis* ATCC 29212 frozen stock from the cryotube using a sterile inoculation loop.
2 Streak *E. faecalis* onto a sterile BHI agar plate.
  Starting from the edge of the Petri dish, in the first quadrant, using the sterile inoculation loop, streak the small chunk of E. faecalis gently over one small section of the agar surface in a zigzag pattern. Using another sterile inoculation loop, drag some bacteria from the last 3 to 5 lines of the zigzag pattern in the first quadrant into the second quadrant. Repeat this streaking process for the third and fourth quadrants using new sterile inoculation loops for each quadrant and dragging from the previous area. This process dilutes the bacteria over the plate to isolate single bacterial colonies.
3 Close the lid and incubate the plate at 36°C for 24 hr in an aerobic atmosphere to obtain isolated and pure colonies of *E. faecalis* (Fig. 5 and 6A).
  Incubate the plate upside down (lid down).

4 Using aseptic technique and a sterile inoculation loop, transfer isolated and pure colonies to a 13 × 100–mm glass tube containing 5 ml sterile BHI broth medium (Fig. 6B) until reaching an optical density of 1.5 at 520 nm (equivalent to 10^9^ CFU/ml) using a spectrophotometer.
  It is important to strictly follow the instructions of the manufacturer of the tube spectrophotometer used.

## Basic Protocol 6 CANAL INOCULATION

Basic Protocol 6 involves inoculating root canals in the sets prepared in Basic Protocol 4.

### Materials

Inoculum (see Basic Protocol 5)
Sets prepared in Basic Protocol 4

Micropipet with sterile micropipet tips
Sterile surface (e.g., sterile microtube lid)
1‐ml syringes with needles, sterile

1 Aspirate 20 µl inoculum into a micropipet with a sterile micropipet tip.
2 Dispense the contents on a sterile surface (e.g., a sterile microtube lid).
3 Aspirate the inoculum using a sterile 1‐ml syringe with needle.
  It is important to avoid air aspiration in this step.
4 Carefully dispense the inoculum inside the root canal in the most apical region (Fig. 6C) in a set prepared in Basic Protocol 4 and immediately close the glass tube of the set containing the inoculated root (Fig. 6D).
  If using the working area of a Bunsen burner, flame the neck of the 5‐ml tube immediately after opening and before closing. This step provides a current that pushes airborne particles away from the opening, reducing the risk of microorganisms entering the tube and therefore preventing contamination.
  Proceed immediately to Basic Protocol 7.

## Basic Protocol 7 SET INCUBATION

This protocol describes how to incubate and maintain the sets throughout all 6 weeks of the experiment. During the incubation process, it is necessary to add sterile fresh medium inside the root canal to guarantee the presence of nutrients and avoid drying out the medium.

### Additional Materials (also see Basic Protocol 6)

Inoculated sets (see Basic Protocol 6)
Enterococcosel broth medium, sterile (see recipe)

36°C bacteriological incubator
Tweezers, sterile
#30 sterile endodontic paper points (TDK)
13 × 100–mm glass tubes

**Figure 7:**
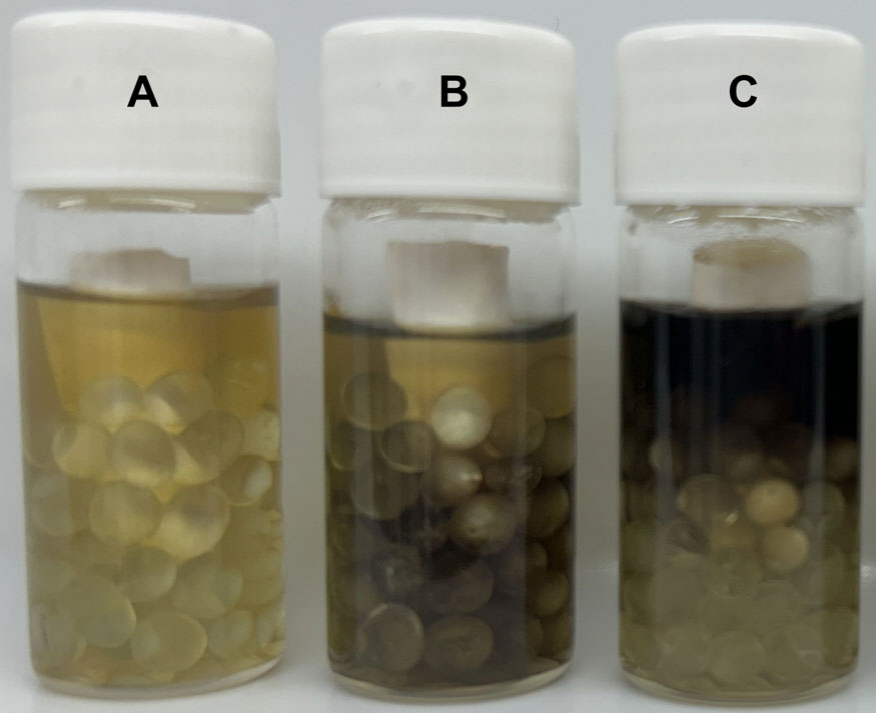
Sets representing the most common results that can be observed. (**A**) Control set (without inoculum), with unchanged color of the bile aesculin agar. (**B**) Microleakage by the canal pathway, with change in the color of the bile aesculin agar to dark brown, initiated from within the medium. (**C**) Surface contamination, with change in the color of the bile aesculin agar to dark brown, beginning at the surface of the medium.

1 Immediately after the inoculation (see Basic Protocol 6), incubate the set at 36°C for 6 weeks in an aerobic atmosphere.
  To avoid leakage of the inoculum to the outside of the tooth, gently open and close the incubator door (this avoids shaking the incubated sets) and keep the sets vertical throughout the experiment. Using a rack adjusted to the diameter of the tubes can help keep them in the vertical position.
2 Forty‐eight hours after inoculation, carefully dispense 20 µl fresh sterile BHI broth medium without inoculum (prepared as in Basic Protocol 6, steps 1 to 3) inside the root canal in the most apical region in the inoculated set.
  It is important to avoid air aspiration in this step.
3 Twice a week, carefully dispense 20 µl fresh sterile BHI broth inside the root canal in the most apical region.
  Steps 2 and 3 ensure the viability of E. faecalis throughout the experiment.
4 Observe all sets daily for 6 weeks to determine if and when bacterial microleakage occurred and by what pathway (Fig. 7).
  No bacterial microleakage results in no color change (Fig. 1, panel I1), similar to what is observed in a non‐inoculated set (Fig. 7A). Change in the bottom portion indicates leakage through the obturation (Fig. 7B), whereas change in the top demonstrates surface contamination (Fig. 7C); in both cases, the set should be discarded.

**Figure 8:**
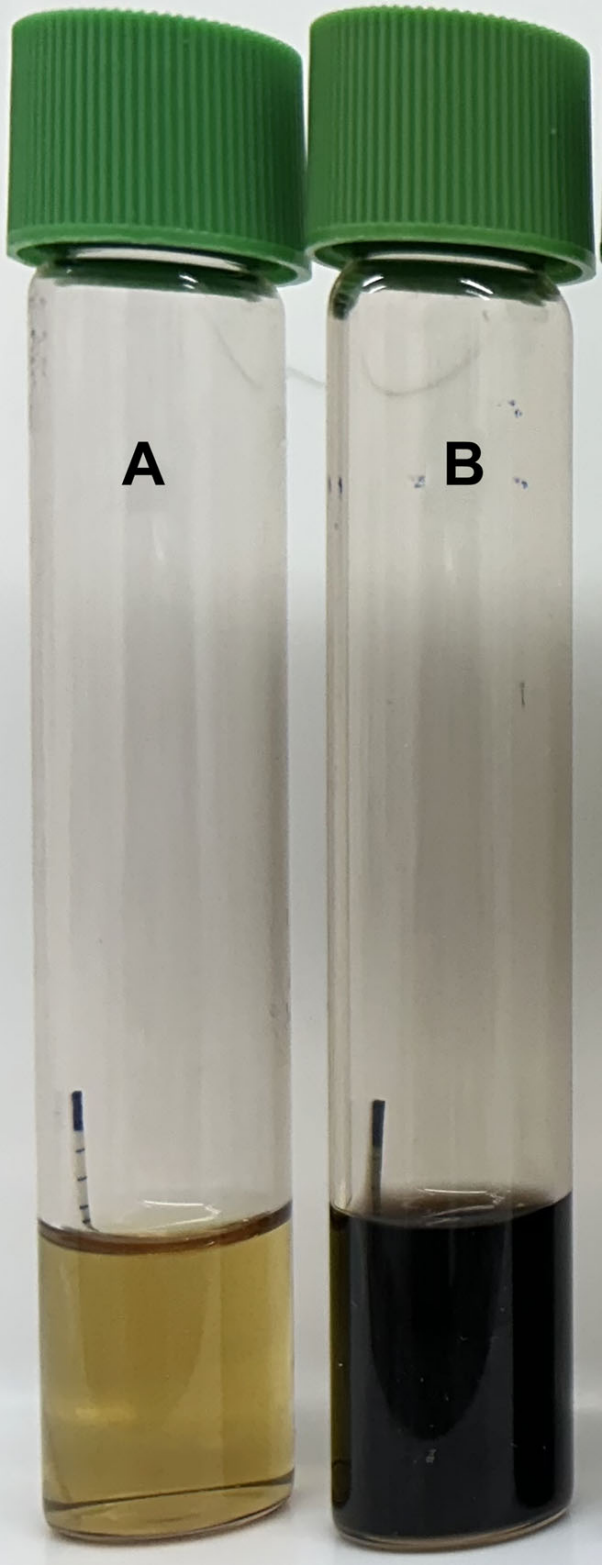
Tubes of enterococcosel broth containing paper points after 24 hr of incubation. (**A**) The medium color remained unchanged (light brown/yellow) because the paper point inserted contained no viable *E. faecalis*. (**B**) The medium color changed to black due to aesculin hydrolysis by viable *E. faecalis* in the broth.

5 After 6 weeks of incubation, using sterile tweezers, insert a #30 sterile endodontic paper point inside the root canals of all sets without bacterial microleakage for 1 min.
6 Transfer the paper point, using sterile tweezers, to a 13 × 100–mm glass tube with 1 ml sterile enterococcosel broth medium.
7 Incubate the tube at 36°C for 24 hr in an aerobic atmosphere.
  Steps 5 to 7 are important to confirm the presence of viable E. faecalis inside the root canals of all sets without color changes. Tubes without color change (Fig. 8A) indicate the absence of E. faecalis in the paper point, whereas tubes with color change (Fig. 8B) indicate the presence of E. faecalis in the paper point. These steps guarantee that the unchanged set color faithfully represents an absence of bacterial microleakage by obturation even with viable E. faecalis inside the root canals.

## REAGENTS AND SOLUTIONS

### BHI agar plates

52 g BHI agar medium powder (Becton, Dickinson and Company, cat. no. BD241830)
Distilled H_2_O to 1000 ml
Combine in 2‐L flask
Stir while heating until all solids are dissolved
Autoclave for 15 min at 121°C
Pour 12 ml into sterile 60 × 15–mm Petri dishes
Allow to solidify at room temperature
Incubate at 36°C for 24 hr to observe any contamination and exclude any contaminated plates
Store ≤3 months at 4°C in sealed bags
  Wait for the BHI agar plates to cool down prior to inoculation with bacteria.

### BHI broth medium

3.7 g BHI broth medium powder (Becton, Dickinson and Company, cat. no. BD237500)
100 ml distilled H_2_O
Combine in 200‐ml flask
Stir until all solids are dissolved
Aliquot 5 ml into 13 × 100–mm tubes with lids
Autoclave for 15 min at 121°C
Store ≤6 months at 4°C
  Ensure that BHI broth medium is sterile before use (sterile BHI broth medium is clear and not cloudy). Wait for the medium to cool down prior to use.

### Bile aesculin agar medium

3.68 g bile esculin agar medium powder (Neogen, cat. no. NCM0117A)
80 mL distilled water
Stir while heating until all solids are dissolved
Autoclave 15 min at 121°C
Allow to solidify at room temperature
Store ≤3 months at 4°C
  Melt in a microwave or boiling water bath prior to use.

### Enterococcosel broth medium

4.3 g bile enterococcosel broth medium powder (Becton, Dickinson and Company, cat. no. BD212207)
100 ml distilled H_2_O
Combine in 200‐ml flask
Stir until all solids are dissolved
Aliquot 1 ml into 13 × 100–mm tubes with lids
Autoclave for 15 min at 121°C
Store ≤6 months at 4°C
  Ensure that bile enterococcosel broth medium is sterile before use (sterile enterococcosel broth medium is clear and not cloudy). Wait for the medium to cool down prior to use.

### Saline, 0.9%

0.9 g sodium chloride
100 ml distilled H_2_O
Autoclave for 15 min at 121°C
Store ≤6 months at 4°C

## COMMENTARY

### Critical Parameters

#### Basic Protocol 1

Considering the anatomical diversity of fresh bovine teeth, expect high exclusion rates and plan to have a larger initial quantity of teeth. It is important to store them in flasks with lids containing sterile 0.9% saline at 4°C to prevent dehydration. The use of a magnifying lens and incident light can improve visualization during selection of bovine teeth with completely formed roots and without visual cracks in the roots. It is important to sharpen the Gracey periodontal curette before removing external teeth structures (e.g., bone, calculus). This protocol should be performed by a dentist or a dental hygienist, depending on the local legislation.

#### Basic Protocol 2

Ensure that the digital phosphor plate is intact and that the intraoral portable X‐ray and the notebook batteries are charged. During X‐rays of the bovine teeth, it is important to fix the teeth in the utility wax to prevent displacement, avoiding distortions in the radiographic images. This protocol must be performed by a trained operator.

#### Basic Protocol 3

For effective decoronation of a bovine tooth, always use a double‐sided diamond cutting disc. It is important to screw the disc completely into the mandrel to avoid it from coming loose during use. Once the disc comes loose, it becomes useless. Keeping teeth hydrated during the cutting process prevents excess dust. After removing the crown, return the root to the refrigerated saline solution.

During canal preparation, secure the bovine root in a bench vise to prevent displacement and charge the endodontic electric motor battery, allowing the operator to perform root canal preparation. Use the endodontic files according to the manufacturer's instructions to avoid their fracture during preparation.

It is important to dry the outer surface of the root to close the apex with utility wax before cleansing of the root canal systems.

To allow the operator to seal the outer surface of the root with nail polish, the use of an object such as a toothpick in the root canal may be helpful. This object can be inserted into a surface like Styrofoam until it is completely dry. To avoid possible adhesion of nail polish to the plastic part of the surgical paper, do not use a tight surgical paper package.

It is important that procedures involving root canals be performed by a specialist in endodontics.

#### Basic Protocol 4

Before inserting glass pearls into the tubes, discard any glass pearls with color other than clear and make sure they are clean and dry, as dark pearls may lead to false‐positive interpretation. A funnel‐like device can be used to assist in inserting the correct amount of glass pearls into the tubes.

Immediately after root canal filling with the test sealer, insert the tooth into the tube within the glass pearls and fill with agar medium to mimic the moist environment of the oral cavity. In this step, it is important to maintain the bile aesculin agar in liquid form, in a water bath between 55° to 60°C, prior to inserting it into the tube. To carry this out, before placing the flask with agar medium in the water bath, the agar medium must be completely melted. To do this, use the agar medium immediately after autoclaving or, if it is stored at 4°C, first melt it in a microwave or in a boiling water bath.

#### Basic Protocol 5

Before streaking *E. faecalis* onto BHI agar plates, ensure the plates with medium are sterile. To do so, incubate them at 36°C for up to 24 hr after preparation to monitor contamination. To cool down the agar plates before inoculating with *E. faecalis*, place them in a refrigerator after removing them from the incubator.

Before transferring colonies to a tube with BHI broth to prepare the inoculum, ensure the broth is sterile and incubate it at 36°C for up to 24 hr after preparation to monitor contamination.

Ensure that the *E. faecalis* on the BHI agar plate is pure. To achieve this, it is important to streak *E. faecalis* to obtain isolated bacterial colonies. Incubate the plate upside down (lid down) and, after the incubation period, carefully observe the morphological characteristics of the colonies. A stereoscopic microscope can aid this observation.

Basic Protocol 5 must be performed by a microbiology‐trained operator, using aseptic technique and sterile tools throughout this protocol.

#### Basic Protocol 6

During inoculation, avoid allowing the inoculum to reach the surface by taking the following precautions: i) when aspirating the inoculum with the syringe needle, avoid aspirating air (bubbles can cause the inoculum to leak onto the bile aesculin agar surface during inoculation process); ii) carefully insert the needle into the canal, ensuring that the inoculum is gently dispensed only into this space; iii) when removing the needle from the canal, do not rest it on the walls to avoid projecting particles containing *E. faecalis* directly onto the bile aesculin agar surface; and iv) keep the sets vertical throughout the experiment. If the inoculum reaches the surface of the bile aesculin agar, the color changes, starting from the surface, indicating contamination (Fig. 7C).

A microbiology‐trained operator must perform this protocol, using aseptic technique and sterile tools during all steps.

#### Basic Protocol 7

During incubation, avoid allowing *E. faecalis* to reach the surface by taking the following precautions: i) the bacteriological incubator door should open and close smoothly, avoiding vibrations and spillage of the inoculum; ii) when adding fresh medium inside the root canal, when aspirating the BHI, avoid aspirating air; ensure that the medium is gently dispensed only into the root canal; and do not rest the needle on the root walls when removing it from the canal; and iii) keep the sets vertical throughout the experiment, avoiding leakage of the bacteria onto the surface of the bile aesculin agar. When the color changes, starting from the surface, it indicates contamination (Fig. 7C).

Basic Protocol 7 must be performed using sterile tools and aseptic technique by a microbiology‐trained operator.

### Troubleshooting

Common problems with the protocols, their causes, and potential solutions are presented in Table 1.

**Table 1:** Troubleshooting Guide for Evaluating Bacterial Microleakage of Endodontic Sealers

Problem	Possible cause	Solution
High rate of teeth exclusion	Anatomical diversity of the teeth	Obtain a larger quantity of teeth than necessary
Tooth dehydration	The teeth were not kept hydrated	Keep the teeth in a saline solution inside flasks with lids and maintain them in the refrigerator at 4°C
Difficult to remove external teeth structures	Blunt Gracey periodontal curette	Sharpen the Gracey periodontal curette on the sharpening stone before removing external tooth structures
Radiographic image showing scratches, alterations, and/or low quality	Scratched or heavily used digital phosphor plate	Replace the digital phosphor plate with a new one
Cutting disc unusable after little use	Insufficient screwing of the disc into the mandrel	Screw the disc completely into the mandrel to prevent it from coming loose during use
Utility wax does not stick to tooth	The bovine tooth is wet	Dry the tooth
Contamination of culture medium	Contamination during pouring the culture medium into the Petri dish and/or during the microbiological process	Perform microbiological steps using aseptic techniques (wear PPE and disinfect gloves and environment surfaces with 70% ethanol) in an aseptic environment such as biosafety cabinet or the working area of a Bunsen burner
Set contamination	Leakage of the medium from the inside of the canal containing *E. faecalis* and/or inoculation outside of the root canal	Avoid bubbles inside the syringe containing inoculum and BHI broth, dispense inoculum or BHI broth only into the canal, avoid projecting particles containing *E. faecalis*, open and close the bacteriological incubator door smoothly, and keep the sets vertical for the duration of the experiment. Train the operator before performing microbiological steps.

### Understanding Results

Through visual selection and mechanical cleaning with a periodontal curette (Fig. 2), Basic Protocol 1 selects a homogeneous group of clean bovine teeth. These selected teeth must not have external structures (e.g., bone, calculus) or cracks. Teeth with fully formed roots with a minimum length of 22 mm are essential for execution of the subsequent protocols. Proper storage in sterile saline solution at 4°C ensures the preservation of the structural integrity of the specimens until use.

Basic Protocol 2, through digital radiographic evaluation, allows the identification of the anatomical characteristics of the root canal. Teeth with multiple canals, lateral canals, or apical deltas should be excluded (Fig. 3A and 3B), and teeth that present a single root canal should be included (Fig. 3C), enabling the use of similar specimens. The decoronation process, chemomechanical endodontic preparation up to #45.06, external sealing of the roots with nail polish, and autoclaving result in a group of sterile 22‐mm bovine roots with isolated external faces, and their single canals are standardized and cleaned at the end of Basic Protocol 3.

Basic Protocol 4 explains the preparation of sterile sets. Each set is composed of a borosilicate glass tube containing glass pearls, an obturated bovine root, and bile aesculin agar (Fig. 4). This basic protocol also describes how to detect possible contamination during set preparation. Contaminated sets should be discarded.

Basic Protocol 5 describes the revival of *E. faecalis* ATCC 29212 from a frozen stock culture. After streaking and incubating at 36°C for 24 hr, isolated colonies should be visible away from the initial streaking area (Fig. 5). Additionally, the preparation of an inoculum from isolated and pure *E. faecalis* colonies (Fig. 6A and 6B) containing 10^9^ CFU per 1 ml BHI broth is presented in this protocol.

The methods described in Basic Protocols 6 and 7 allow us to obtain inoculated sets (Fig. 6C and 6D) with 20 µl (∼2 × 10^7^ CFU/ml) *E. faecalis* inside the root canal, incubated vertically for 6 weeks, with addition of fresh medium inside the root canals twice a week and daily observation to detect changes in the color of the bile aesculin agar (Fig. 7). Figure 7 represents the most common results that can be observed in this protocol: i) unchanged color of the medium (bile aesculin agar), resulting from the absence of bacterial microleakage by the endodontic sealer used to obturate the root canals, similar to what is observed in the non‐inoculated set (Fig. 7A); ii) change in the color of the bile aesculin agar from yellow/light brown to dark brown, initiated from within the medium due to the hydrolysis of aesculin by *E. faecalis*, resulting from the presence of bacterial microleakage through the endodontic sealer used to obturate the root canals (Fig. 7B); and iii) change in the color of the bile aesculin agar from yellow/light brown to dark brown, beginning at the surface of the medium, due to aesculin hydrolysis by *E. faecalis*, resulting from the presence of this bacterium on the agar surface, indicating contamination via the external root pathway. Sets presenting the characteristics in ii) or iii) should be discarded (Fig. 7C).

At the end of the assay (6 weeks), in sets without color change (similar to Fig. 7A), the presence of viable *E. faecalis* in the root canal needs to be confirmed. To do this, a sterile paper point can be inserted into the root canal and transferred to a broth that indicates the presence of *E. faecalis*. After the incubation period, esculetin, resulting from the hydrolysis of aesculin, reacts with ferric ions present in the medium, such as enterococcosel broth, forming a dark complex (Fig. 8B). Conversely, the medium maintains its original color in the absence of the microorganisms that cause this reaction (Fig. 8A).

**Figure 9:**
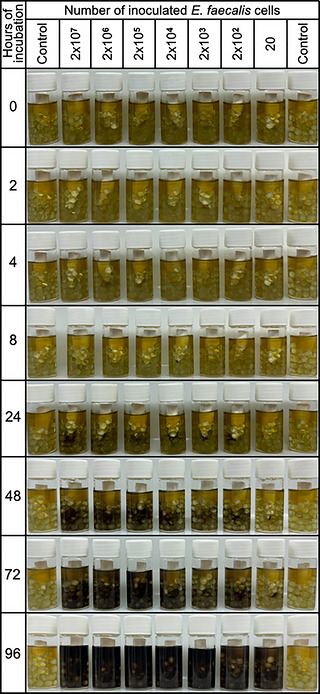
Different concentrations of *E. faecalis* inoculated inside the root canal over time.

**Figure 10:**
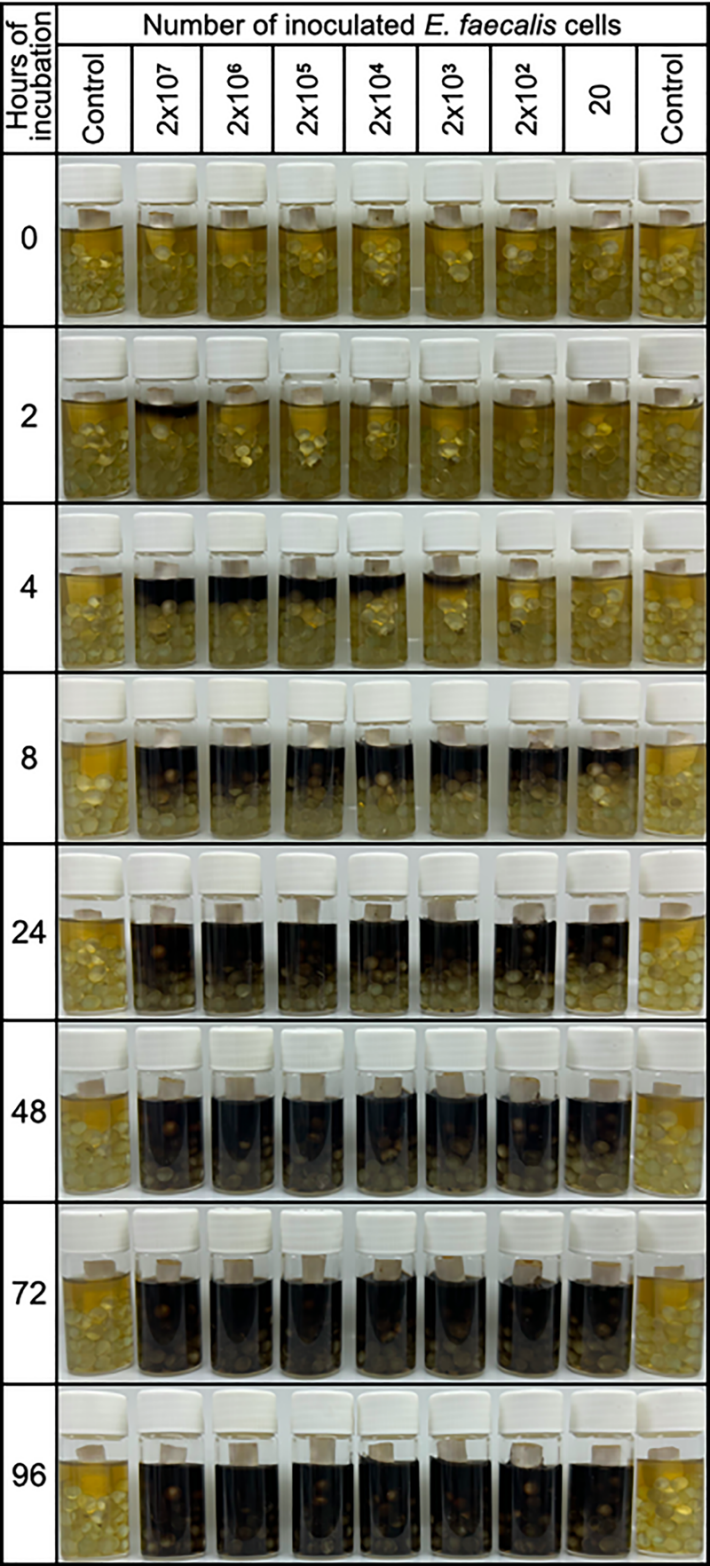
Different concentrations of *E. faecalis* inoculated externally to the root (on the surface of the medium) over time.

Figures 9 and 10 show different concentrations of *E. faecalis* over time, inoculated either inside the root canal without obturation or externally to the root, respectively. The concentration of the inoculum is inversely proportional to the time required for the medium to change color, regardless of where the inoculation occurs. Comparing these figures, there is a clear difference when *E. faecalis* reaches the culture medium through the root canal (Fig. 9; color is first seen at the bottom and disseminates from the bottom upward) or through contamination on its surface (Fig. 10; color is first seen at the top and spreads downward), regardless of the inoculum concentration. Therefore, the tubes contaminated on the surface can be easily identified during daily visual inspection.

**Figure 11:**
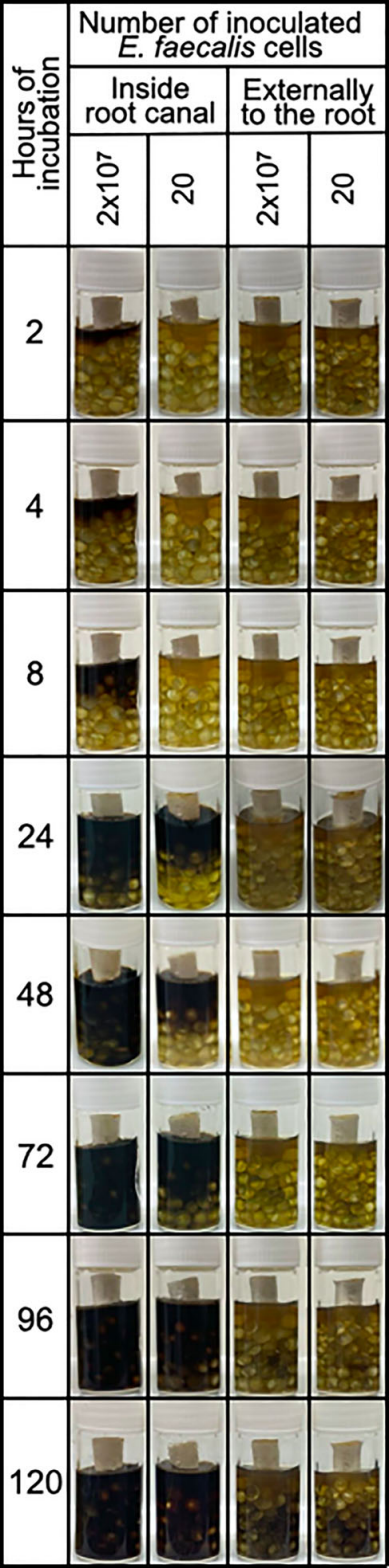
Time course for different concentrations of *E. faecalis* inoculated internally and externally to the root (on the surface of the medium) in sets previously incubated for 6 weeks at 36°C without inoculation.

Figure 11 demonstrates the viability of the set throughout the experiment. After 6 weeks of incubation without inoculation, during which fresh medium was introduced into the root canal twice a week, the canals were inoculated with *E. faecalis*. As shown in the figure, despite a slight delay, the set allows for the detection of microbial presence and enables the distinction between intra‐ and extracanal inoculation.

It is important to note that sets maintained in the incubator for 6 weeks without the addition of fresh medium exhibited signs of dehydration. This characteristic indicates that the supplementation with fresh medium within the root is essential not only for nourishing the microorganisms but also for preventing dehydration of the medium present in the set.

Despite the advantages of the proposed approach, some limitations inherent to *in vitro* bacterial leakage models should be considered. Although the methodology was designed to improve visualization of the inoculum infiltration pathway, *in vitro* endodontic models are unable to completely mimic the complexity of clinical conditions and host‐microorganism interactions observed *in vivo* (Swimberghe et al., 2019). Previous reviews have demonstrated that the clinical relevance of *in vitro* endodontic bacterial microleakage studies remains controversial and may be influenced by methodological variability among studies (Abdin & Al‐Tayyan, 2023; Savadkouhi et al., 2016; Snigdha & Karobari, 2025).

Another limitation is the use of a monospecies inoculum. Even though *E. faecalis* is the most commonly used microorganism in endodontic laboratory studies (Oishi et al., 2026; Swimberghe et al., 2019), monospecies models do not completely reproduce the diversity and ecological interactions presented *in vivo* because endodontic biofilms are multispecies, and their morphologic structure may vary from case to case, with no unique pattern identified for endodontic infections (Ricucci & Siqueira, 2010; Siqueira et al., 2010).

In addition, although bovine teeth are considered a suitable first‐choice substitute for human teeth in research due to the similarity between the chemical composition of human and bovine enamel and dentin (de Dios Teruel et al., 2015), their use may influence bacterial penetration and leakage. Furthermore, variability related to specimen preparation, tooth morphology, inoculum standardization, incubation conditions, and operator handling may affect reproducibility across different laboratory settings.

As this is a novel approach proposed to improve the visualization and control of the bacterial infiltration pathway, comparisons with other microleakage assessment methods have not yet been reported in the literature. Therefore, future studies comparing this approach with other endodontic leakage assays, including dye penetration, fluid filtration, and microbial leakage in two‐chamber models, may be useful to further validate its reproducibility, sensitivity, and clinical relevance. Finally, although this proposed laboratory method for evaluating bacterial microleakage of endodontic sealers may provide valuable comparative information regarding their sealing ability, caution should be exercised when extrapolating laboratory findings to clinical conditions.

### Time Considerations

The entire set of protocols can be completed in approximately 8 to 9 weeks, but depending on slaughterhouse availability as well as the number of roots tested, the period can be considerably extended.

The selection and cleaning of bovine teeth (Basic Protocol 1) take 1 day. Basic Protocol 2 requires 1 day to take and analyze the radiographs, applying the inclusion criteria. The preparation of the teeth included in the study (Basic Protocol 3) is the most laborious phase of teeth preparation (Basic Protocols 1 to 3). It begins with the decoronation of the bovine teeth, which takes 1 to 2 days, and the standardization of the cervical, middle, and apical thirds of the teeth can be performed in 1 to 2 days. Root canal cleaning takes 1 day, but an additional day (for complete drying) is required before performing the next step. Sealing the tooth with enamel takes 2 days. Basic Protocol 4 needs about 2 to 3 days to prepare the sets containing glass pearls, bovine root with its canal filled with endodontic sealer, and solid culture medium in a glass tube with a lid. One day is required between set preparation and its inoculation. Basic Protocols 5 and 6 require 2 days to prepare the medium, streak the *E. faecalis*, prepare the inoculum, and perform the canal inoculation. Basic Protocol 7 (set incubation) requires daily observation for 6 weeks, with addition of fresh medium inside root canals twice a week.

### Author Contributions

**Emannuel Fonseca Thomé da Silva** Monteiro: Conceptualization; investigation; methodology; visualization; writing—original draft; writing—review and editing. **Alice Abib Ramos Fabri**: Conceptualization; methodology; writing—review and editing. **Marcelo Barbosa** Garcia: Conceptualization; methodology; visualization; writing—review and editing. **Sabrina Silva** Azevedo: Conceptualization; methodology; visualization; writing—review and editing. **Higor Machado** Gonçalves: Conceptualization; methodology; visualization; writing—review and editing. **Ana Clara Miranda Porto** Figueiredo: Conceptualization; methodology; visualization; writing—review and editing. **Fabrícia Nogueira** Klein: Investigation; methodology; visualization; writing—review and editing. **Gabriela Ceccon** Chianca: Investigation; methodology; visualization; writing—review and editing. **Roberta** Barcelos: Conceptualization; methodology; visualization; writing—review and editing. **Helvécio Cardoso Corrêa** Póvoa: Investigation; visualization; methodology; writing—review and editing. **Leonardo Santos** Antunes: Conceptualization; investigation; methodology; visualization; writing—review and editing. **Natalia Lopes Pontes Póvoa** Iorio: Conceptualization; funding acquisition; investigation; methodology; project administration; resources; supervision; visualization; writing—original draft; writing—review and editing.

### Conflict of Interest

All authors have no conflicts of interest to declare.

## Data Availability

Data sharing is not applicable to this article as no new data were created or analyzed in this study.
